# The Effect of Visual Impairment on Postural Stability After Lumbar Spine Fracture: A Case Report and Review of the Literature

**DOI:** 10.7759/cureus.49995

**Published:** 2023-12-05

**Authors:** Fahad Alhelal, Sami AlEissa, Majed Abaalkhail, Abdullah Alshehri, Abdullah Alsaeed, Joud Bindekhayel

**Affiliations:** 1 Department of Medicine, King Abdulaziz Medical City, Ministry of the National Guard Health Affairs, Riyadh, SAU; 2 Department of Surgery, Unaizah College of Medicine and Medical Sciences, Qassim University, Riyadh, SAU; 3 College of Medicine, Al Imam Mohammad Ibn Saud Islamic University, Riyadh, SAU

**Keywords:** adult spine deformity, postural instability, spinal deformity, spinal-fusion, visual impairment

## Abstract

The role of vision in maintaining postural stability is crucial, and its loss, whether congenital or acquired, can significantly impact sensory-motor interactions, leading to musculoskeletal abnormalities and defective gait patterns. This case report discusses the complex interplay between visual impairment, post-traumatic kyphosis, and the development of spinal deformity in a 79-year-old blind patient. The patient sustained a simple fall resulting in an L1 compression fracture in 2016. Despite conservative treatment for the fracture, progressive spinal deformity became evident both clinically and radiographically. Further assessments, including evaluation of bone healing, facet arthroplasty, disc degeneration, and canal compromise, were performed. The patient's altered gait and postural abnormalities were indicative of the impact of visual impairment on postural stability. After addressing osteoporosis through endocrinology consultation and medical management, the patient underwent posterior spinal instrumentation and deformity correction, leading to a successful post-operative recovery with a return to baseline functional status.

Visual impairment disrupts postural stability by limiting sensory input and prompting compensatory mechanisms, which may increase postural sway and instability. This abnormal gait further contributes to spinal deformities, and the fear of falling can exacerbate postural instability, limiting mobility. Over time, persistent postural imbalance leads to the creation of a state of continual asymmetric stress related to the spinal axis, which can progress to the development of spinal deformities, creating a self-perpetuating cycle.

This case underscores the significance of vision in postural stability and the adverse effects of visual impairment on spinal alignment. The development of spinal deformities in visually impaired individuals, especially in the presence of risk factors like osteoporosis, emphasizes the need for early intervention and postural training to prevent irreversible deformities. Decisions regarding surgical or non-surgical interventions for spinal deformities in visually impaired patients must consider multiple factors, including clinical symptoms, appearance, pain, functional limitations, and social issues. Future research should explore effective interventions for improving postural stability in visually impaired individuals and preventing the development of spinal deformity.

## Introduction

Humans primarily use vision to enable the brain to assess information regarding the relative position of the body in space and adjust the posture accordingly [1]. Congenital or acquired loss of vision causes improper sensory and motor interactions, which result in typical musculoskeletal abnormalities, that subsequently lead to defective gait patterns, causing disabilities [2]. Postural stability is the body's ability to maintain balance; it is frequently tested by postural sway, which is defined as continual deviation and correction of the center of gravity (COG) on a very limited base of support [3,4]. The absence of vision causes impairments in posture due to insufficient contact with the environment, disrupts standard gait and balance patterns, and increases the tendency to fall [1]. In order to compensate for faulty gait, postural abnormalities further develop creating a "vicious cycle" as suggested by research that mechanical forces imposed by an asymmetric loading directly cause the structures of the spinal column to be deformed once the spinal curvature is triggered [1]. These anomalies subsequently give rise to an additional level of fixed asymmetric loading, which perpetuates the development [5,6]. To maintain equilibrium, the gait of a blind person deviates from normal, which leads to defective body mechanics. This is achieved naturally by compensation in the development of posture [1].

Adult spinal deformity (ASD) is becoming increasingly prevalent in the elderly population. ASD can arise from an integration of factors, including aging, decreasing bone density, and atypical posture, which as suggested in this research can be brought on by vision impairment. Although there may be several etiologies, symptoms are associated with asymmetrical and progressive degeneration of the facet joints, discs, and other spinal components that may cause compression of the neural elements [7]. Spinal deformity is defined as a curvature in the spine where the alignment is outside of defined normal limits [7]. A surgical intervention decision can be based on clinical symptoms, appearance, pain, functional restrictions, and social issues. These weigh up against the potential risks and limitations of the suggested surgical procedure [8]. Osteoporosis can cause vertebral body collapse, resulting in rapid progression of spinal deformity. The degree of spinal deformity increases due to stress on the weakened vertebral bodies. This weakening or inadequate spine support may also contribute to the severity of the symptoms associated with the spinal deformity [7].

Due to the lack of evidence supporting the link between the absence of vision and the propagation of spine deformity, we present this case to hypothesize a relationship of cause and effect between them. Further research should be done to emphasize the need for early intervention in visually impaired individuals and postural training to prevent irreversible deformities.

## Case presentation

**Figure 1:**
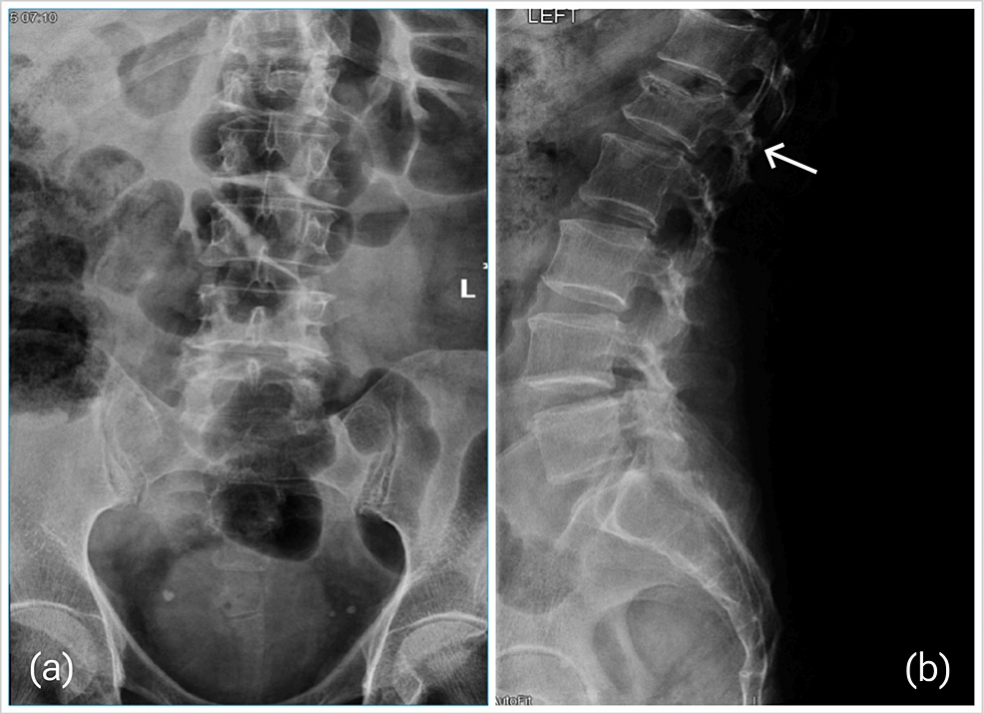
X-ray scans upon presentation. (a) AP view and (b) lateral view, which illustrates L1 compression fracture with no signs of significant kyphosis or focal malalignments in the coronal and sagittal planes

In this case report, we discuss a 79-year-old blind patient with a history of recurrent falling, who sustained a simple fall back in 2016. The fall resulted in an L1 compression fracture, and the decision at that time was to treat the patient conservatively (Figure 1).

**Figure 2:**
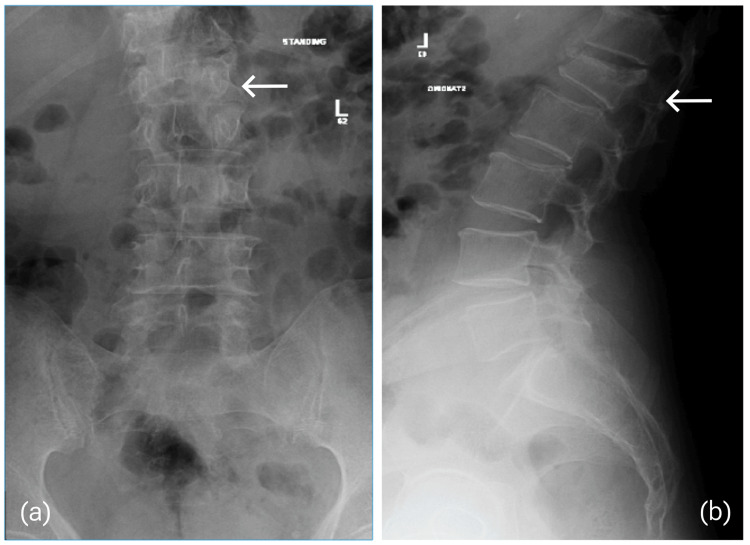
X-ray scan four weeks after presentation. (a) AP view that suggests increasing right truncal shift in the coronal plane, and (b) lateral view that shows a developing focal kyphosis

The patient continued to follow up in the outpatient clinic with regular x-ray imaging upon visits (Figure 2).

**Figure 3:**
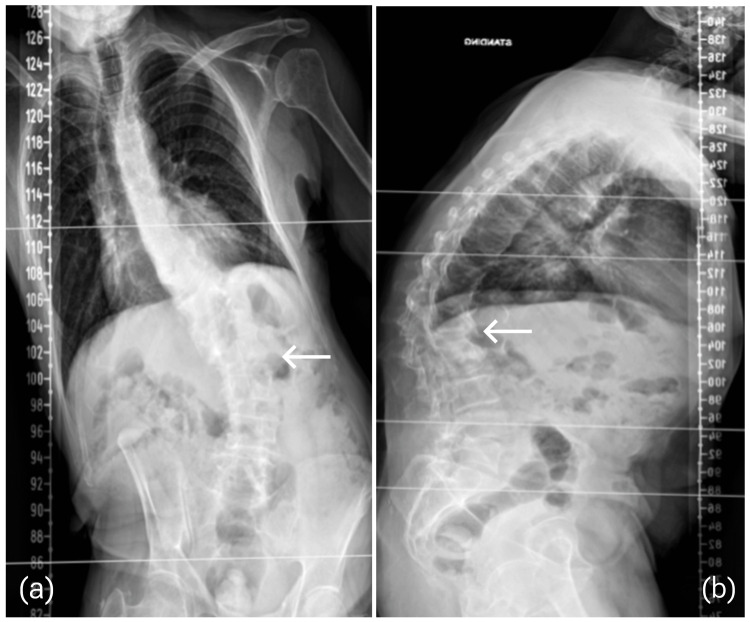
Scoliosis view x-rays show (a) AP view of a severe coronal right-sided shift with focal malalignment at L1 vertebrae and (b) lateral view demonstrating positive sagittal imbalance

The fracture showed signs of healing; however, there was an increasing deformity clinically and radiographically on the coronal and sagittal planes (Figure 3).

**Figure 4:**
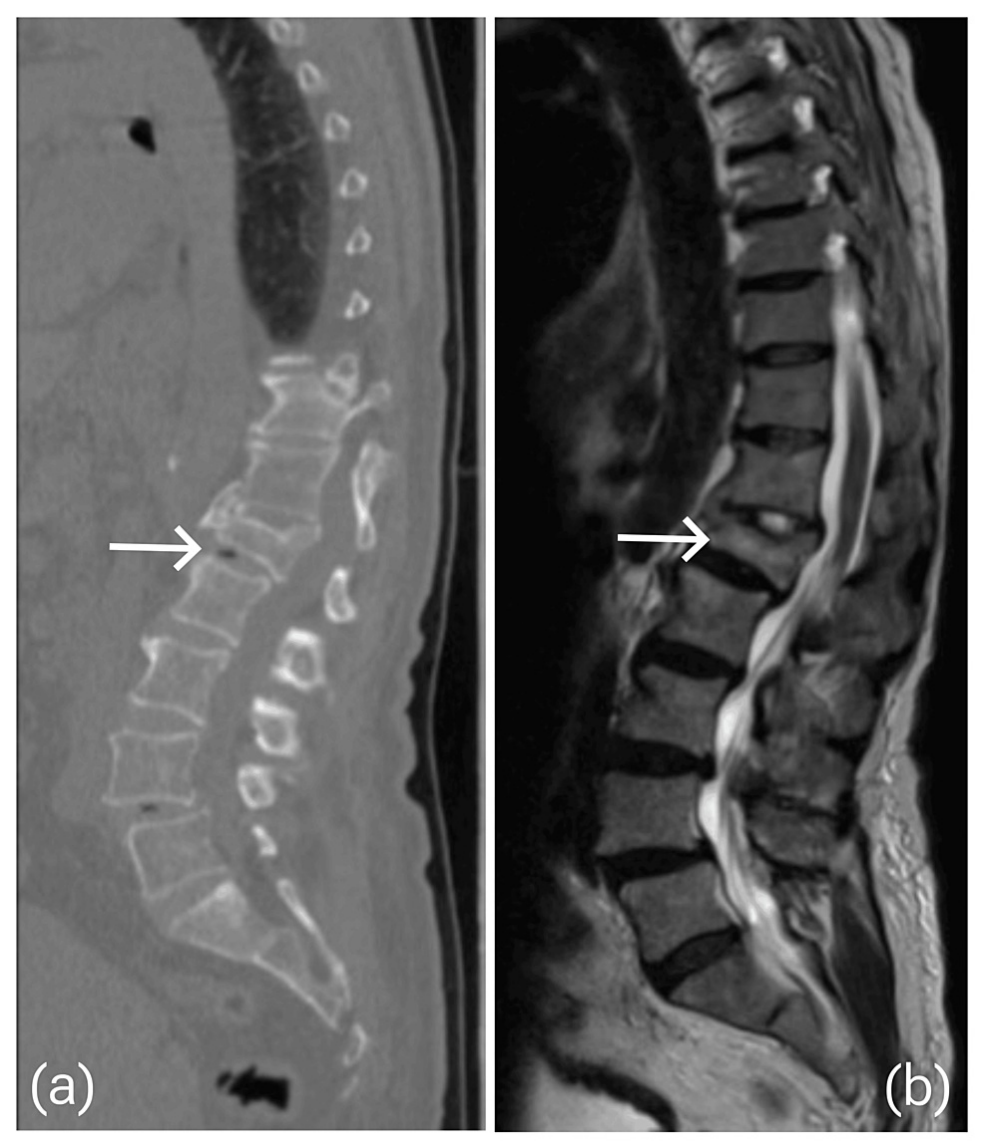
(a) CT scan showed old healed compression fracture at L1 with more than 50% vertebral height loss and moderate scoliotic deformity with the apex at L1. (b) MRI T2 weight image shows L1 healed compression fracture and upper lumbar scoliotic deformity with no thecal sac compromise and multiple degenerative disc disease

Further imaging investigations were done to evaluate the bone healing, facet arthroplasty, disc degeneration, and canal compromise (Figure 4).

Upon examination of the patient, he had an altered gait with the tendency to fall forward and lean to his right side. The patient complained of tenderness on his thoracolumbar region upon palpation; however, his neurological examination was unremarkable with good motor power, no loss of sensation, and no signs of motor neuron lesion.

After investigation, the dual x-ray absorptiometry (DEXA) scan showed a T-score of -3.2 indicating osteoporosis. We consulted endocrinology to manage his osteoporosis with medications; the patient received anabolic anti-osteoporosis for a period of 18 months. Operative management was offered to the patient after treating his osteoporosis.

The patient underwent posterior spinal instrumentation and deformity correction. The post-operative period was uneventful with a gradual return to his baseline functional status.

To our knowledge, there are no studies demonstrating the effect of blindness on the development of post-traumatic coronal or sagittal imbalances in visually impaired patients. In this case report we show the patient’s presentation, pre-operative follow up, management, and post-operative outcomes.

Our aim is to report a case of blindness and post-traumatic kyphosis that led to degenerative scoliosis and global spinal imbalance and to review the literature on the effect of visual impairment over postural balance to hypothesize the role of vision impairment in the propagation of spine deformity.

Surgical procedure

Pre-operatively, the patient’s condition was optimized in preparation for the operative management. After obtaining informed consent, the patient was brought to the operating room and placed in the prone position on the operating table. General anesthesia was induced without complications. The back was prepped and draped in a sterile fashion.

A midline incision was made from T4 to S2. The paraspinal muscles were then dissected off the spine bilaterally. With the spine exposed, segmental spinal instrumentation was then placed from T4 to S2. Pedicle screws were inserted at each level under fluoroscopic guidance. Multiple Ponte osteotomies were performed at the pre-determined vertebral levels, at the apex of the kyphotic deformity to the patient’s overall sagittal and coronal imbalances. Hemostasis was maintained throughout the procedure. Each screw was tightened to the rod, ensuring the spine was held in the correct alignment. Following the placement of the instrumentation and achieving the desired correction, autograft and allograft bone were placed along the decorticated posterior elements to promote fusion. The wound was irrigated thoroughly. Hemostasis was ensured. The fascia was closed with absorbable sutures. The subcutaneous tissue was approximated, and the skin was closed using a running subcuticular suture. A sterile dressing was applied.

**Figure 5:**
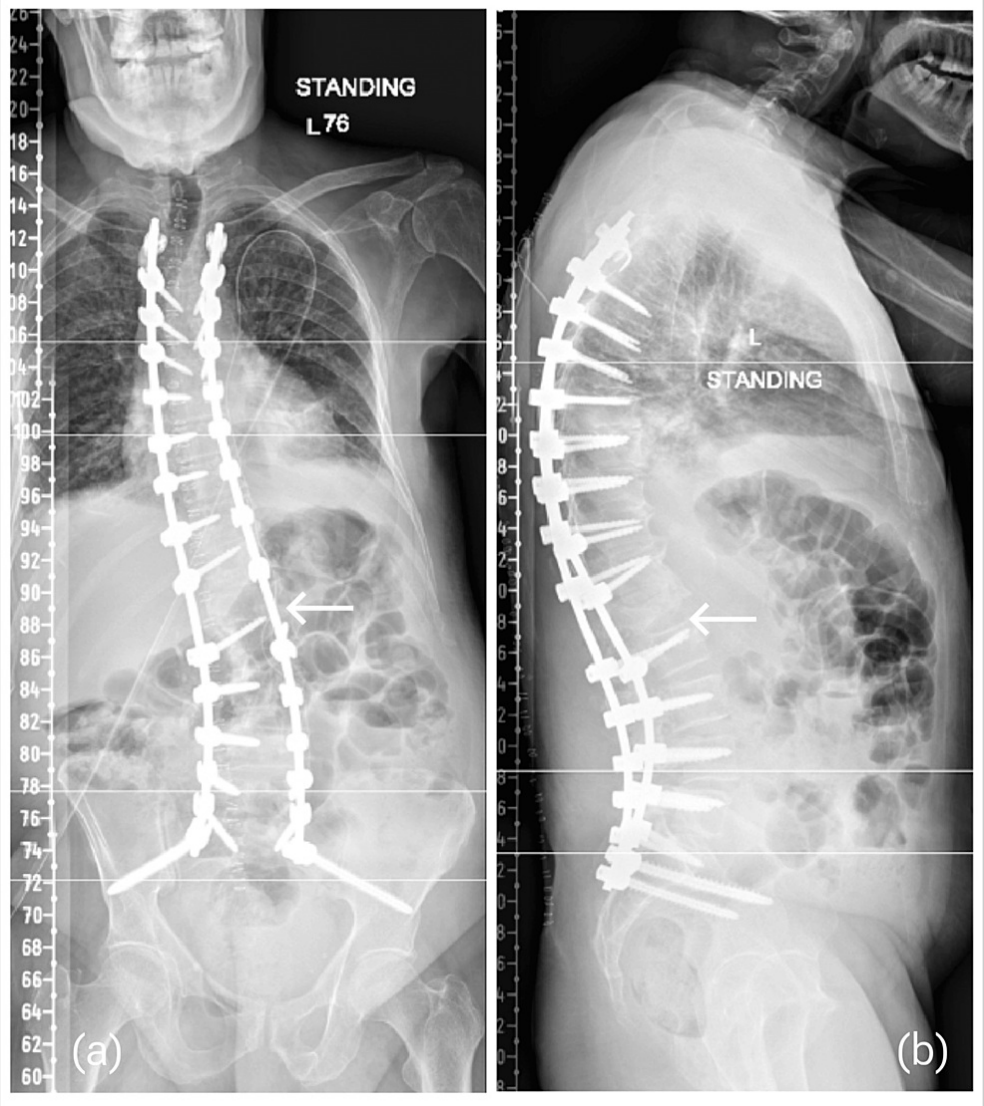
Post-operative scoliosis view x-rays display posterior spinal instrumentation from T4 to S2. (a) AP view shows correction of coronal alignment, and (b) lateral view shows correction of sagittal alignment

Post-operatively, the patient was started on standard pain management medication, prophylactic blood thinners, and antibiotics. He tolerated surgery well without complications and with a significant improvement in his pre-operative pain and functional level. No problem was observed with wound healing. Full spine precautions were followed in our patient at all times, and he was instructed to follow them when he was discharged on day 10. Six months after surgery, x-rays demonstrated intact instrumentation with a maturing osseous fusion. No complications occurred secondary to the reaming procedure (Figure 5).

## Discussion

Postural stability is a complicated process that requires the integration of sensory input from multiple sources, including the visual, vestibular, and somatosensory (proprioceptive) systems [9]. Visual input contributes significantly to postural control by providing information about the position and movement of the body relative to the environment. Before initiating a movement, spatial concepts and their application are essential to maintain a base of support and COG [10]. Individuals with visual impairments often rely on compensatory mechanisms to maintain postural stability. These mechanisms may include increased reliance on somatosensory and vestibular inputs to establish patterns of movement and positions in space (efficient movement) [11]. However, these compensatory strategies may not be as effective as visual input, leading to increased postural sway and instability [10,11]. Also, their standing balance is impaired because vision loss impacts the vestibular system by interfering with feedback from the visual system [4]. Consequently, visually impaired individuals develop an abnormal gait to maintain equilibrium including retraction of the head, increased pelvic rotation, excessive backward leaning of the trunk with dorsal kyphosis, compensating forward head posture, abnormal contralateral trunk and arm movements, and flexion contractures leading to deficient body mechanics [1].

This manifests as a significant obstacle to older adults, with visual impairments particularly, because the loss of balance and mobility becomes a barrier to independence that is associated with the fear of falling [12]. This fear can further exacerbate postural instability, as individuals tend to adopt more cautious and rigid postural strategies, limiting their range of motion and flexibility. This defensive approach to posture may also increase the risk of falls and restrict overall physical activity levels [11]. Moreover, people with postural instability are at higher risk of developing spinal deformities such as scoliosis or kyphosis, as persistent postural imbalance can lead to the creation of a state of continual asymmetric stress relative to the spinal axis over time [5]. Recent research supports the long-held theory that spinal deformity is caused directly by postural imbalance, regardless of the initial cause, because the dynamics within vertebrae are affected by persistent asymmetric mechanical pressure [6]. The resultant spinal deformity and the asymmetrical loading create a "vicious cycle" that typically repeats itself, as it indicates that once a spinal curvature is triggered and continuous asymmetric loading develops, the mechanical forces imposed by asymmetric loading directly cause structures of the spinal column to be deformed. Such abnormalities, in turn, generate a new degree of fixed asymmetric loading, leading to continuing progression [5,6].

ASD is a common condition in spine clinics with various presentations affecting the thoracic or thoracolumbar spine throughout aging [7]. ASD can be caused by multiple etiologies, but symptoms usually present due to the progressive asymmetric degeneration of spinal components that leads to the compression of neural elements [13]. The condition starts, irrespective of the primary cause, with progressive asymmetric degeneration that evolves into an imbalance of the structural support of the spinal column, characterized by an abnormal curvature in the sagittal plane (kyphosis, lordosis) creating an imbalance in the patient's front or back or the coronal plane (scoliosis) causing imbalance to the patient's right or left side [7,13]. One condition that is commonly associated with ASD is osteoporosis; it occurs when there is decreased bone formation with increased or continued bone resorption leading to decreased bone mass overall [7]. In a more advanced setting, osteoporosis can lead to a collapse in vertebral bodies and may accelerate and further exacerbate ASD [7,14]. 

Ideally, postural training and re-education of the body’s pattern of reaction and coordination should be started in people with visual impairment and postural instability before developing irreversible deformities [1,2]. This can be attained by sensory input reinstating either by available sensory systems like hearing for analyzing orientation or using a prosthesis. Blind people’s rehabilitation focuses majorly on the “perceptual contact” with the ground using a sensory tool like a long cane. After the link with the external environment is made, postural training can be started to enhance mobility skills [1].

After developing deformities, there is no definite algorithmic management for ASD [15]. Previously, surgeons relied on conservative ASD treatment options due to higher perioperative morbidity and a high incidence of neurological impairments linked with older age group operational management. However, surgical intervention became the standard of ASD treatment, because of the improvement in surgical techniques, the raised consideration of the importance of spinopelvic alignment, and owing to the failure of medical treatment to improve the health status of ASD patients [15]. Yet, non-surgical intervention is still widely considered, as some research supports it where it results in improvement in patients with ASD [8]. Accordingly, the decision between operative and nonoperative treatment for ASD might be difficult for surgeons and patients where benefits are weighed against the potential risks and limitations related to the proposed surgical treatment [15]. Decisions are made based on multiple factors like progressive deformity, pain, cosmesis, the magnitude of potential intervention, clinical symptoms, appearance, pain, functional limitations, and social issues [8].

## Conclusions

In conclusion, this case emphasizes the significance of vision in maintaining postural stability. In addition, it shows the effect of visual impairment in developing spinal deformities, particularly in longstanding unstable posture related to continuous asymmetrical loading on the spine accompanied by risk factors like old age and osteoporosis. Future studies should examine efficient methods for enhancing postural stability in those who are visually impaired for the prevention of spinal deformity formation.
